# Impact of simulated patient-based communication training vs. real patient-based communication training on empathetic behaviour in undergraduate students – a prospective evaluation study

**DOI:** 10.1186/s12909-024-05801-8

**Published:** 2024-08-12

**Authors:** Vanessa Britz, Jasmina Sterz, Yannik Koch, Teresa Schreckenbach, Maria-Christina Stefanescu, Uwe Zinßer, Rene Danilo Verboket, Katharina Sommer, Miriam Ruesseler

**Affiliations:** 1 Goethe University Frankfurt, Medical Faculty, Institute for Medical Education and Clinical Simulation, Frankfurt/Main, Germany; 2 Goethe-University Frankfurt, University Hospital, Department of Trauma Hand and Reconstructive Surgery, Frankfurt/Main, Germany; 3 Goethe University Frankfurt, University Hospital Frankfurt, Department of General, Visceral, Transplantation and Thoracic Surgery, Frankfurt/Main, Germany; 4 Johannes Gutenberg University, Medical Center Mainz, Department of Pediatric Surgery, Mainz, Germany

**Keywords:** Simulated patient, Communication training, Empathy, Undergraduated, Medical eduction

## Abstract

**Background:**

Empathy is a key competency and is essential for doctor-patient relationships. Studies have proven a continuous reduction of empathy in medical students during their study period. The use of SPs is positively evaluated for competency acquisition and real patient communication training has positive effects on empathy empowerment. Therefore, the present study focusses on the impact of simulated patient (SP) vs real patient (RP) communication training on empathetic behaviour in undergraduate medical students.

**Methods:**

The prospective evaluation took place during a 210-minute skills lab unit on medical communication for 3rd year medical students. Study participants were allocated in advance to one of three groups: one group trained with an SP (SP-group) and was informed about the fact that it was an SP; another group trained with an SP but assumed to encounter an RP (incognito patient group (IP-group)); the last group trained with an RP and was correctly informed about it (real patient group (RP-group). Self-assessed empathy was measured by using Jefferson Scale of Physician Empathy (JSPE) and Interpersonal Reactivity Index (IRI), as these are the most commonly used instruments for assessing empathy. Study participants were evaluated on empathetic behaviour by their group-associated patient using the Consultation and Relational Empathy (CARE) scale.

**Results:**

146 students participated. There was no significant difference in self-assessed empathy between groups for JSPE and IRI. External assessment via CARE showed a statistically significant difference between SP-group and IP-group , as well as between SP-group and RP-group. There was no significant difference between IP-group and RP-group. This means that students training with real patients (or who believed them to be real) did receive significantly lower performance ratings on their empathy.

**Conclusion:**

The results demonstrate a significant lower external empathy rating for students who had trained with a real patient or if they were in the belief of having encountered a real patient; this may be due to inhibitions and a lack of routine. Therefore, we recommend implementing SPs in the early study period with the gradual integration of RPs in the student’s further course of study.

## Background

Studying medicine combines scientific curiosity with social and altruistic aspects. On the other hand, medical studies are traditionally oriented towards scientific content, so soft skills such as empathy and communication are scarcely emphasised and happen to be neglected in modern medicine [1–4]. In recent years, approaches and changes have already been developed to better address empathy and communication in medical teaching. These include, for example, longitudinal communication curricula, implementation guidelines and training of breaking bad news [5–8]. Nevertheless, there is still a need for improvement.

Empathy is a key competency for every doctor, regardless of the field of expertise, since the most frequent medical tasks are conversations with patients and their next of kin [9–11]. The empathy shown by the doctor is essential for a good doctor-patient relationship, while it also facilitates diagnostics and, therefore, has an impact on therapeutical outcomes [12, 13]. Rakel et al. showed that patients with a common cold had significantly shorter convalescence times if a high level of empathy was displayed by their treating physician; white blood cell counts (neutrophile granulocytes) and levels of interleukin 8 also decreased according to the subjective perception of the patients [14]. However, the development of empathy plays a role not only for the doctor-patient relationship but also for personal progress and mental health, since there are indications that in situations where medical student empathy is high, burnout is highly likely to be low [15].

Despite its undeniable importance, studies have proven that there is a continuous reduction of empathy in medical students during their study period [2, 3]. This is especially the case in young doctors; after starting regular patient contact, their empathy levels seem to drop. Potential reasons for this might include the high workload, high emotional stress levels and other stressors associated with entering the profession [2, 3, 16, 17].

Many countries such as Canada and the United States have already reacted to this perception by adjusting their curricula, naming empathy as an educational goal [18, 19]. In Germany, the National Competency-Based Catalogue of Learning Objectives Medicine, a nationwide standardised catalogue of learning targets, also includes empathy and communication skills, as do the sub-catalogues of certain medical fields [20]. The use of SPs has already been positively evaluated in terms of competency acquisition [21], while Ahrweiler et al. have shown that using real patients for student training and a patient-centered education also have positive effects on empathy empowerment [22].

The present study aims to compare directly the impact that simulated patient-based communication training vs. real patient-based communication training has on the empathetic behaviour in undergraduate students.

## Methods

### Participants and background

The present study is a prospective evaluation of the impact that simulated patient-based communication training has on empathetic behaviour in medical students.

It was performed according to the ethical principles of the World Medical Association Declaration of Helsinki (Ethical Principles for Medical Research Involving Human Subjects) and was reviewed by the ethical committee of the University Hospital. As stated by the ethical committee, no further approval was required.

Study participants were undergraduate medical students in their 3rd year of studies. At this stage the majority of the students had minimum to no experience with SP training. The study took place during the mandatory surgical skills lab training to the extent of one week, which was then followed by a surgical internship of two weeks’ duration. The prerequisite for attending the skills lab and the internship was the completion of the surgical main lecture course and passing the associated written exam.

Participation in the study took place after detailed oral and written explanations and consent. Furthermore, participation in the study was voluntary and could be terminated at any time without disclosing reasons.

### Communication unit

The skills lab contains 12 teaching units for basic practical medical skills, one of which is a communication training unit that instructs for correct medical history taking and the meaningful structuring of informed consent discussions for surgical interventions. This unit of 210 min duration is held by peer tutors who have been specifically trained beforehand. The quality of the units is monitored and maintained by using tutor manuals, standardised presentations and mandatory tutor training on a regular basis.

Besides the medical content of the informed consent discussions, the unit focusses on communicative competencies such as empathetic behaviour and strategies to deal with taboo-afflicted topics. It contains theoretical parts as well as practical training. First, the tutors and students work out the headings on medical history taking and informed consent discussions and discuss the required contents. Afterwards, the students practise via role-playing exercises followed by a 360° feedback on content-related aspects and social interactions with the patient. By the end of the unit, each student has taken part in at least one history taking training or has led at least one informed consent discussion. The setting of the role-playing exercise is a hospital one, either at the ward or in the emergency room, in which the students act either in the role of a young doctor or an experienced final-year student, depending on the scenario.

### Empathy measurement

To evaluate empathy within a study a valid and objective tool is required. An overview on accessible tests is given by Hemmerdinger et al. [23]. For self-assessment of empathy, the Jefferson Scale of Physicians Empathy (JSPE) and the Interactivity Index (IRI) are the most common ones and these were used in the present study. The JSPE was developed especially for people with medical backgrounds, whereas the IRI is a more general measure [24, 25]. While the JSPE has its unique features, it yields significant overlap with dimensions of empathy that are relevant to patient care (perspective taking and empathic concern) of the IRI. A statistically significant correlation of a moderate magnitude exists between the total scores of the JSPE and IRI [26].

The JSPE is a standardised questionnaire with 20 items; it evaluates self-assessed empathy and the relevance of empathy for medical personnel in a medical context. This questionnaire, established by Hojat et al., is validated and especially designed for people with a medical background [24]. Questions are answered on a 7-point Likert scale, ranging from “1 = do not agree” to “7 = fully agree”. There are questions that aim for empathy as well as those that query being non-empathetic. Therefore, the latter questions have to be reversed in polarity for evaluation.

The Interpersonal Reactivity Index (IRI) was developed by Davis et al. and contains 28 items that are rated on a 5-point Likert scale [25]. The scale ranges from “1 = strongly disagree” to “5 = highly agree”. As in the JSPE, not all questions query for being empathetic and have to be repolarised before evaluation.

In the present study, the Consultation and Relational Empathy (CARE) scale, designed by Mercer et al. especially for rating medical conversational situations by the patient, was used [27]. This is categorised as an external assessment tool for empathy rated by an involved person and contains 10 items, rated on a 5-point Likert scale, ranging from “1 = fully applies” to “5 = don’t apply at all”. The question design leads to a low score if the experienced empathy was subjectively high.

### Intervention

Epidemiologic data of each participant were gathered before the start of the unit. Moreover, participants were asked to fill in the JSPE and the IRI, and each generated an individual code to ensure an anonymous data collection.

Prior to the skills lab, the students were assigned to their respective groups by the Office of the Dean. The assignment took place unbiased by the principal investigator and regardless of study participation. Due to the curricular integration of the study, an individual randomisation was not possible.

According to their group allocation, the students attended the communication unit. One group undertook the role-play training with a simulated patient (SP-group) and was informed about the fact that it was an SP. Another group trained with a simulated patient but assumed that they were encountering a real patient, since both the students and the tutors had been informed so by the principal investigator (incognito patient group (IP-group)). The last group trained with a real patient and were correctly informed about this, as well as their tutors (real patient group (RP-group)). The theoretical parts and the time limits did not differ between the three groups.

The study participants were evaluated in terms of empathetic behaviour immediately after taking a medical history or an informed consent discussion with their group-associated patient using the CARE scale. All patients (SPs, IPs, RPs) were recruited from our trained SP-pool and were trained on how to use the CARE scale. When performing as SPs (SP-group) or IPs (IP-group) the patients adhered to a standardised, scripted role completely different from their personal history. As RPs (RP-group), the patients had no scripted role but reported on their own personal medical and social histories. This guaranteed that all participating assessors all had the same level of experience with the students.

They were also pre-selected according to the presence of current or past surgical conditions so that they could draw from their own experience as RPs.

All sheets were marked with the individual code of the student and gathered in a closed envelope. The evaluation only took place at the end of term to avoid recognition and linking of the sheets to the respective student.

### Statistical methods

Data were processed using Microsoft Excel (Microsoft Inc., Redmond, WA, USA). Statistical analysis was performed using IBM SPSS 24 (SPSS Inc., Chicago, IL, USA) and measurement of the effect size was performed using G*Power (University Düsseldorf, Düsseldorf, Germany).

Tests between the groups were calculated using parametric variant analysis ANOVA.

If variant homogeneity was found, the p-values for comparing the groups were analysed using the Tukey test. In cases of variant heterogeneity, a corrected ANOVA (Welch’s t-test) was used and for p-value analyses between groups, the Games-Howell test was applied. If the data was normally distributed, the analysis of differences between the sexes and the prior training of the participants was performed by using the student’s t-test for independent samples. If not normally distributed, the data were analysed using the Kolmogorov-Smirnov test for independent samples. The effect size was distributed according to Cohen’s d, based on the means and standard deviations.

## Results

### Epidemiologic data

A total of 189 students attended the skills lab during the study period and 146 gave consent for study participation; of these, 38.4% were males, the median age being 22.9 ± 2.8 years. The study population was, therefore, representative of a 3rd year undergraduate semester at Frankfurt Goethe University Hospital. While gathering the epidemiologic data, participants were asked to provide information about their pre-educational experience. To simplify the data, these were clustered into fields: medical and/or social field (e.g. nurse, paramedic), other pre-educational experience, and none. There were no significant differences within the three study cohorts.

### Results of empathy measurement

#### Self-assessed empathy

Table 1 demonstrates the results of the self-assessment via the JSPE and IRI subscales. The higher the score, the higher was the empathetic self-assessment of the student. There were no statistically significant differences between the groups within the items of the subscales.

**Table 1:** Self-assessment via JSPE and IRI

Scale	Items	Group	*N*	Mean	SD	Min	Max	*p*
JSPE	Perspective Taking	SP	46	57.61	5.825	42	67	0.177
		IP	44	54.91	6.765	35	70	
		RP	51	55.94	7.893	36	69	
	Compassionate Care	SP	49	45.16	4.069	35	53	0.817
		IP	44	45.27	5.415	31	55	
		RP	51	45.82	6.784	23	55	
	Walking in Patient Shoes	SP	48	8.10	2.176	4	12	0.444
		IP	44	8.75	2.771	3	14	
		RP	53	8.47	2.358	2	14	
IRI	Perspective Taking	SP	49	24.53	4.686	12	33	0.544
		IP	43	24.05	4.525	10	33	
		RP	51	25.51	4.204	15	33	
	Fantasy	SP	49	25.47	4.805	14	33	0.108
		IP	43	23.05	5.411	12	33	
		RP	52	24.48	6.060	10	35	
	Empathic Concern	SP	49	26.49	4.510	16	34	0.801
		IP	43	26.79	4.149	15	34	
		RP	52	27.06	4.146	13	34	
	Personal Distress	SP	47	16.15	3.759	9	25	0.107
		IP	43	16.12	3.995	8	24	
		RP	51	17.61	4.065	8	25	

(SP = simulated-patient-group; IP = incognito-patient-group; RP = real-patient-group)

#### External assessment of empathy

Table 2 displays the assessment results of the students by the group-associated patient using the CARE scale. The maximum score is 5, and the minimum 1. In this case, the lower the score, the more empathetic the student was rated by the group-associated patient.

**Table 2:** External assessment using the CARE-Scale

Group	SP	IP	RP	Total
*N*	49	44	53	146
Mean	1.65	2.33	2.14	2.04
Standard-Deviation	0.59	0.77	0.77	0.76
Minimum	1	1	1	1
Maximum	4	4.7	3.7	4.7

(SP = simulated-patient-group; IP = incognito-patient-group; RP = real-patient-group)

**Fig. 1:**
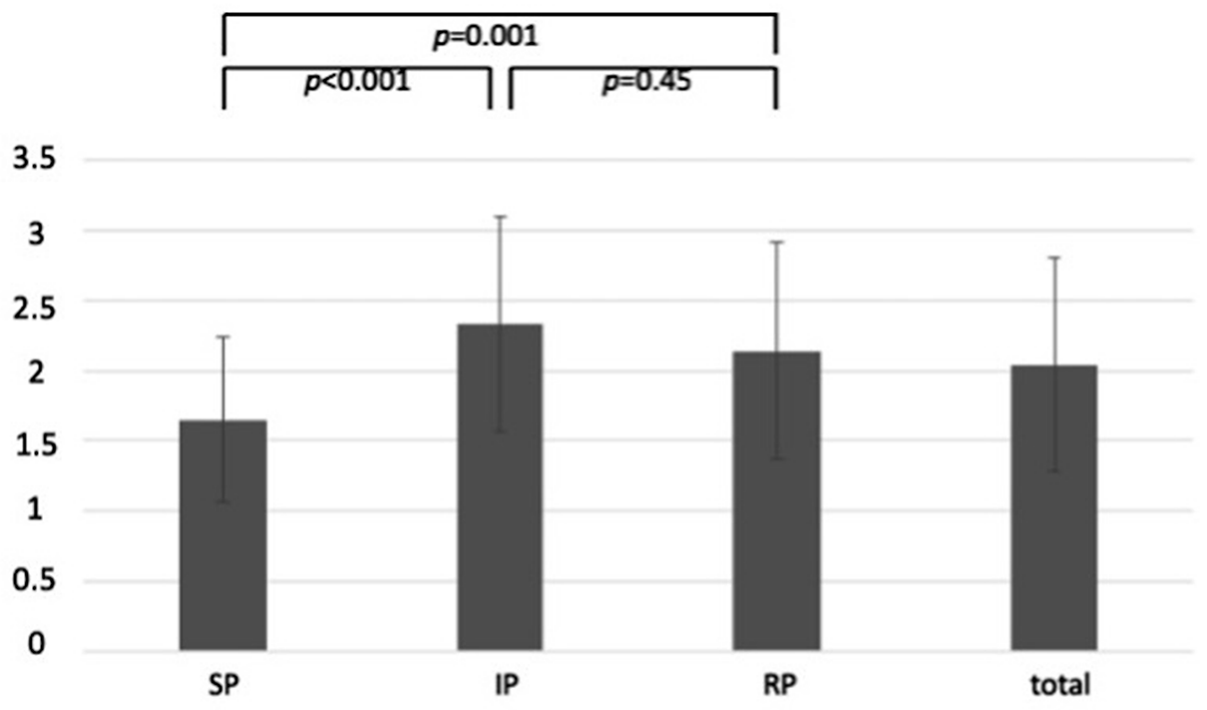
External assessment using the CARE-Scale (SP = simulated-patient-group; IP = incognito-patient-group; RP= real-patient-group). Y-axis displays CARE-Scale rating. Data are presented as mean + standard deviation

The results show a significant difference between SP-group and IP-group (*p* < 0.001; Cohen’s d: 0.99), as well as between SP-group and RP-group (*p* = 0.001; Cohen’s d: 0.71), both of which have a high effect size. There was no significant difference between IP-group and RP-group (*p* = 0.45; Cohen’s d: 0.25). Accordingly, students who trained with real patients (or who believed the patients to be real) were rated as less emphathetic (i.e. they had a significantly higher score on the CARE scale). [Figure 1 near here]

## Discussion

Our results demonstrate a significantly lower external empathy rating for students if they had trained with a real patient or were in the belief of having encountered a real patient when training. This might seem surprising at first when taking into account former published results on this topic. Ahrweiler et al. showed that using real patients for student training and a patient-centred education have positive effects on empathy empowerment [22]. Nevertheless, many studies prove a significant decrement of empathy over the medical training period [2, 3, 16, 17]. Hojat et al. described the beginning of bedside training as the time with the highest decrement in empathy [28]. This also seems to be a crucial aspect of the present study. The skills lab training is placed at the beginning of the clinical medical training, therefore, the communication unit is in many cases the first real patient encounter for the students. The lack of routine and, as a result, the accompanying stress may have contributed to inhibitions on the student’s side while working with a real patient, thus, leading to the lower ratings.

It is already known that higher stress levels can lead to lower personal performance. A review by LeBlanc et al. concluded that stress is a main impairment factor in terms of memory, multi-tasking and decision making, although how a person handles stress is a highly individual process [29].

The use of SPs as well as RPs has already been positively evaluated in terms of competency acquisition [21, 22]. Based on the present study, it seems important to plan consciously the occupation of SPs and RPs within the curriculum blueprint regarding the development of communication skills, especially empathy training. Using SPs in the early study period could create a safe space to attune the students to patient encounters and to lower their initial existing inhibitions. Subsequently, as the students’ knowledge develops and having acquired advanced skills, RPs should be gradually integrated into their training. However, further studies are needed to define in more detail the transition period from SP to RP.

The implementation of (simulated) patient’s feedback to foster empathetic behaviour development seems to be beneficial. The SP’s feedback to students has been found to be positively evaluated and appreciated, [30, 31] thus, some faculties already rely on the assessment of SPs [32]. These positive effects might facilitate the gain of confidence and development of empathy in the early phases of the students’ training.

Another reason for lower empathy rates in the RP-group could arise from the personal rating tendencies of the assessor. Assessments by real patients can be stricter due to a higher personal involvement and, therefore, hold higher expectations than the assessment of an SP. This phenomena is known as the hawk-dove effect and describes different rating tendencies in different persons that are mostly due to biographical differences [33]. One can assume that a real patient, talking about their own, often emotionally afflicted history, demands higher requirements from the student’s empathy than an SP who is not emotionally involved. Under this assumption, however, one would also have expected a difference between IP- and RP-group. There are various possible explanations for this. One might be that the students possibly demonstrate a higher level of empathy in known testing scenarios with the SP but with a real or perceived real patient, thus correspondingly less practice artificiality, they begin to forget they are being tested and start to show a more intuitive behavior. Fuller et al. showed in an interview based study that one factor that influences empathy competence is the hierarchical position, since communicators often “communicate from their place in the hierachy“ [34]. The subjective perception of this position may shift for a student between an SP and an RP, which might contribute to a change in empathy.

On the side of the assessors, the specificity of the selected RPs, who were also all trained SPs, may play a role. Simmenroth-Nayda et al. conducted an interview study on the effects of being an SP on real patient life [35]. They found them to be more attentive, having a better understanding of the circumstances under which doctors work and to act more self-confidently. This insider knowledge may have led to a weakening of the biographical hawk-dove effect and thus milder assessments as a real patient. Further studies and replications in the future may help to better assess this aspect.

A noteworthy limitation of this study is the narrow focus, determined by just one type of skills training unit within one semester period. However, the study includes the results of nearly a whole cohort of medical students at the faculty without selectional bias, and, therefore, is still eligible for making valid deductions.

There are numerous studies that analyse the impact that student’s personality traits and emotion recognition abilities have on their demonstrated empathy [36]. Another interesting aspect would be an evaluation of the patient’s own personality traits and empathy levels and their impact on the assessment. This aspect was not covered in this study and should, therefore, be subject to further research.

## Conclusion

Our results demonstrate a significant, lower external empathy rating for students if they had trained with a real patient, or were in the belief of having encountered a real patient when in training; this low empathy rating was possibly due to their inhibitions and lack of routine. Based on these data, the thesis can be put forward that it could be beneficial to impelent SPs in the early study period with the gradual integration of RPs in the further course of communication training and empathy development. However, further exploration of the observed mechanisms is needed.

## Data Availability

Data is provided within the manuscript. The datasets used and/or analysed during the current study are available from the corresponding author on reasonable request.

## Abbreviations

CARE: Consultation and Relational Empathy
IRI: Interpersonal Reactivity Index
JSPE: JEFFERSON Scale of Physician Empathy
RP: Real patient
SP: Simulated patient
